# *N*-(4-Chloro­phenyl­sulfon­yl)-2,2,2-tri­methyl­acetamide

**DOI:** 10.1107/S1600536808017583

**Published:** 2008-06-19

**Authors:** B. Thimme Gowda, Sabine Foro, B. P. Sowmya, P. G. Nirmala, Hartmut Fuess

**Affiliations:** a Department of Chemistry, Mangalore University, Mangalagangotri 574 199, Mangalore, India; b Institute of Materials Science, Darmstadt University of Technology, Petersenstrasse 23, D-64287 Darmstadt, Germany

## Abstract

In the crystal structure of the title compound (N4CPSTMAA), C_11_H_14_ClNO_3_S, the conformations of the N—H and C=O bonds in the amide group are *anti* to each other, similar to those observed in *N*-phenyl­sulfonyl-2,2,2-trimethyl­acetamide (NPSTMAA) and 2,2,2-trimethyl-*N*-(4-methyl­phenyl­sulfon­yl)acetamide (N4MPSTMAA). The bond parameters in N4CPSTMAA are similar to those in NPSTMAA, N4MPSTMAA, *N*-aryl-2,2,2-trimethyl­acetamides and 4-chloro­benzene­sulfonamide. The –SNHCOC– unit including the amide group is essentially planar and makes a dihedral angle of 82.2 (1)° with the benzene ring, comparable to the values of 79.1 (1) and 71.2 (1)° in NPSTMAA and N4MPSTMAA, respectively. The mol­ecules in N4CPSTMAA are linked into a chain by inter­molecular N—H⋯O hydrogen bonds.

## Related literature

For related literature, see: Gowda *et al.* (2003 ▶, 2007 ▶, 2008**a* ▶,b* ▶).
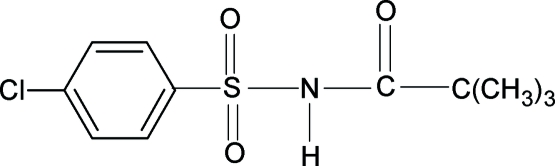


## Experimental

### 

#### Crystal data

C_11_H_14_ClNO_3_S
*M* *_r_* = 275.74
Triclinic, 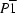

*a* = 6.034 (2) Å
*b* = 10.695 (2) Å
*c* = 11.134 (2) Å
α = 67.13 (2)°
β = 79.76 (2)°
γ = 88.46 (2)°
*V* = 650.8 (3) Å^3^
*Z* = 2
Mo *K*α radiation
μ = 0.45 mm^−1^
*T* = 299 (2) K
0.50 × 0.24 × 0.12 mm

#### Data collection

Oxford Diffraction Xcalibur diffractometer
Absorption correction: multi-scan (**CrysAlis RED**; Oxford Diffraction, 2007 ▶) *T*_min_ = 0.806, *T*_max_ = 0.948
7016 measured reflections
2595 independent reflections
1901 reflections with *I* > 2σ(*I*)
*R*_int_ = 0.023

#### Refinement

*R*[*F* ^2^ > 2σ(*F* ^2^)] = 0.042
*wR*(*F* ^2^) = 0.129
*S* = 1.10
2595 reflections
157 parameters
H atoms treated by a mixture of independent and constrained refinement
Δρ_max_ = 0.24 e Å^−3^
Δρ_min_ = −0.51 e Å^−3^

### 

Data collection: *CrysAlis CCD* (Oxford Diffraction, 2007 ▶); cell refinement: *CrysAlis RED* (Oxford Diffraction, 2007 ▶); data reduction: *CrysAlis RED*; program(s) used to solve structure: *SHELXS97* (Sheldrick, 2008 ▶); program(s) used to refine structure: *SHELXL97* (Sheldrick, 2008 ▶); molecular graphics: *PLATON* (Spek, 2003 ▶); software used to prepare material for publication: *SHELXL97*.

## Supplementary Material

Crystal structure: contains datablocks I, global. DOI: 10.1107/S1600536808017583/is2303sup1.cif

Structure factors: contains datablocks I. DOI: 10.1107/S1600536808017583/is2303Isup2.hkl

Additional supplementary materials:  crystallographic information; 3D view; checkCIF report

## Figures and Tables

**Table 1:** Hydrogen-bond geometry (Å, °)

*D*—H⋯*A*	*D*—H	H⋯*A*	*D*⋯*A*	*D*—H⋯*A*
N1—H1*N*⋯O2^i^	0.82 (3)	2.19 (3)	2.986 (3)	165 (3)

Symmetry code: (i) 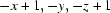.

**Fig. 1.**
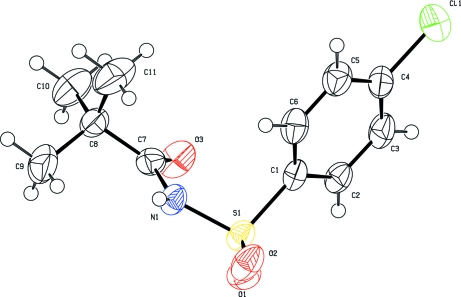
Molecular structure of the title compound, showing the atom labeling scheme. The displacement ellipsoids are drawn at the 50% probability level. H atoms are represented as small spheres of arbitrary radii.

**Fig. 2.**
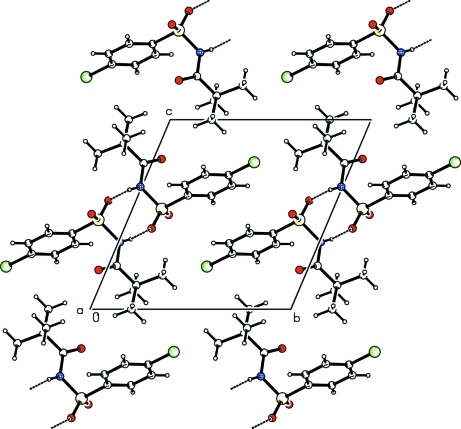
Molecular packing of the title compound with hydrogen bonding shown as dashed lines.

## supplementary crystallographic information

### Comment

In the present work, as part of a study of the substituent effects on the solid state geometries of *N*-(aryl)-sulfonamides and substituted amides, the structure of *N*-(4-chlorophenylsulfonyl)-2,2,2-trimethylacetamide (N4CPSTMAA) has been determined (Gowda *et al.*, 2003, 2007, 2008*a,b*). The conformations of the N—H and C=O bonds of the SO_2_—NH—CO—C group in N4CPSTMAA are *anti* to each other (Fig. 1), similar to those observed in *N*-(phenylsulfonyl)-2,2,2-trimethylacetamide (NPSTMAA) and (4-methylphenylsulfonyl)-2,2,2-trimethylacetamide (N4MPSTMAA) (Gowda *et al.*, 2008*a,b*). The bond parameters in N4CPSTMAA are similar to those in NPSTMAA, N4MPSTMAA, *N*-(aryl)-2,2,2-trimethylacetamides (Gowda *et al.*, 2007) and 4-chlorobenzenesulfonamide (Gowda *et al.*, 2003). The packing diagram of N4CPSTMAA molecules showing the hydrogen bonds N—H···O (Table 1) involved in the formation of molecular chains is shown in Fig. 2.

### Experimental

The title compound was prepared by refluxing 4-chlorobenzenesulfonamide with excess pivalyl chloride for about an hour on a water bath. The reaction mixture was cooled and poured into ice cold water. The resulting solid was separated, washed thoroughly with water and dissolved in warm sodium hydrogen carbonate solution. The title compound was precipitated by acidifying the filtered solution with glacial acetic acid. It was filtered, dried and recrystallized from ethanol. The purity of the compound was checked by determining its melting point. It was characterized by recording its infrared and NMR spectra. Single crystals of the title compound were obtained from an ethanolic solution and used for X-ray diffraction studies at room temperature.

### Refinement

The N-bound H atom was located in a difference map and its positional parameters were refined, with *U*_iso_(H) = 1.2*U*_eq_(N). The refined N—H length is 0.82 (3) Å. The other H atoms were positioned with idealized geometry (C—H = 0.93–0.96 Å) and were refined  using a riding model, with *U*_iso_(H) = 1.2*U*_eq_(C).

### Crystal data

**Table:**

C_11_H_14_ClNO_3_S	*Z* = 2
*M**_r_* = 275.74	*F*_000_ = 288
Triclinic, *P*1	*D*_x_ = 1.407 Mg m^−^^3^
Hall symbol: -P 1	Mo *K*α radiation λ = 0.71073 Å
*a* = 6.034 (2) Å	Cell parameters from 2338 reflections
*b* = 10.695 (2) Å	θ = 2.3–27.9º
*c* = 11.134 (2) Å	µ = 0.45 mm^−^^1^
α = 67.13 (2)º	*T* = 299 (2) K
β = 79.76 (2)º	Long needle, colourless
γ = 88.46 (2)º	0.50 × 0.24 × 0.12 mm
*V* = 650.8 (3) Å^3^	

### Data collection

**Table:**

Oxford Diffraction Xcalibur diffractometer	2595 independent reflections
Radiation source: fine-focus sealed tube	1901 reflections with *I* > 2σ(*I*)
Monochromator: graphite	*R*_int_ = 0.023
*T* = 299(2) K	θ_max_ = 26.4º
φ and ω scans	θ_min_ = 2.3º
Absorption correction: multi-scan(*CrysAlis RED*; Oxford Diffraction, 2007)	*h* = −6→7
*T*_min_ = 0.807, *T*_max_ = 0.948	*k* = −13→13
7016 measured reflections	*l* = −13→13

### Refinement

**Table:**

Refinement on *F*^2^	Secondary atom site location: difference Fourier map
Least-squares matrix: full	Hydrogen site location: inferred from neighbouring sites
*R*[*F*^2^ > 2σ(*F*^2^)] = 0.042	H atoms treated by a mixture of independent and constrained refinement
*wR*(*F*^2^) = 0.129	*w* = 1/[σ^2^(*F*_o_^2^) + (0.0498*P*)^2^ + 0.5579*P*] where *P* = (*F*_o_^2^ + 2*F*_c_^2^)/3
*S* = 1.10	(Δ/σ)_max_ = 0.001
2595 reflections	Δρ_max_ = 0.24 e Å^−^^3^
157 parameters	Δρ_min_ = −0.51 e Å^−^^3^
Primary atom site location: structure-invariant direct methods	Extinction correction: none

### Special details

**Table:**

Geometry. All e.s.d.'s (except the e.s.d. in the dihedral angle between two l.s. planes) are estimated using the full covariance matrix. The cell e.s.d.'s are taken into account individually in the estimation of e.s.d.'s in distances, angles and torsion angles; correlations between e.s.d.'s in cell parameters are only used when they are defined by crystal symmetry. An approximate (isotropic) treatment of cell e.s.d.'s is used for estimating e.s.d.'s involving l.s. planes.
Refinement. Refinement of *F*^2^ against ALL reflections. The weighted *R*-factor *wR* and goodness of fit *S* are based on *F*^2^, conventional *R*-factors *R* are based on *F*, with *F* set to zero for negative *F*^2^. The threshold expression of *F*^2^ > σ(*F*^2^) is used only for calculating *R*-factors(gt) *etc*. and is not relevant to the choice of reflections for refinement. *R*-factors based on *F*^2^ are statistically about twice as large as those based on *F*, and *R*- factors based on ALL data will be even larger.

### Fractional atomic coordinates and isotropic or equivalent isotropic displacement parameters (Å^2^)

**Table:**

	*x*	*y*	*z*	*U*_iso_*/*U*_eq_	
C1	0.3219 (4)	0.2479 (2)	0.6007 (2)	0.0368 (5)	
C2	0.2137 (4)	0.3650 (2)	0.5986 (2)	0.0407 (6)	
H2	0.0845	0.3889	0.5615	0.049*	
C3	0.3008 (5)	0.4452 (3)	0.6522 (3)	0.0477 (7)	
H3	0.2315	0.5244	0.6509	0.057*	
C4	0.4906 (5)	0.4074 (3)	0.7075 (3)	0.0471 (6)	
C5	0.5980 (5)	0.2899 (3)	0.7113 (3)	0.0501 (7)	
H5	0.7249	0.2650	0.7504	0.060*	
C6	0.5129 (4)	0.2108 (3)	0.6559 (3)	0.0461 (6)	
H6	0.5841	0.1326	0.6558	0.055*	
C7	0.0583 (4)	−0.0409 (3)	0.7704 (2)	0.0394 (6)	
C8	0.0948 (5)	−0.1803 (3)	0.8733 (2)	0.0438 (6)	
C9	0.0658 (8)	−0.2896 (3)	0.8210 (4)	0.0875 (13)	
H9A	0.1730	−0.2718	0.7408	0.105*	
H9B	−0.0842	−0.2892	0.8032	0.105*	
H9C	0.0903	−0.3768	0.8861	0.105*	
C10	−0.0751 (6)	−0.2068 (4)	1.0003 (3)	0.0711 (10)	
H10A	−0.2252	−0.2043	0.9821	0.085*	
H10B	−0.0538	−0.1382	1.0333	0.085*	
H10C	−0.0535	−0.2945	1.0654	0.085*	
C11	0.3316 (6)	−0.1784 (4)	0.9012 (3)	0.0791 (11)	
H11A	0.3474	−0.1092	0.9344	0.095*	
H11B	0.4390	−0.1595	0.8209	0.095*	
H11C	0.3583	−0.2652	0.9661	0.095*	
N1	0.1921 (4)	−0.0075 (2)	0.6461 (2)	0.0408 (5)	
H1N	0.291 (5)	−0.055 (3)	0.630 (3)	0.049*	
O1	0.0027 (3)	0.1924 (2)	0.49806 (19)	0.0521 (5)	
O2	0.3885 (4)	0.13680 (19)	0.42621 (17)	0.0554 (5)	
O3	−0.0693 (3)	0.0397 (2)	0.79256 (19)	0.0574 (5)	
Cl1	0.60248 (18)	0.50755 (8)	0.77505 (9)	0.0778 (3)	
S1	0.21651 (11)	0.14675 (6)	0.52882 (6)	0.0413 (2)	

### Atomic displacement parameters (Å^2^)

**Table:**

	*U*^11^	*U*^22^	*U*^33^	*U*^12^	*U*^13^	*U*^23^
C1	0.0414 (13)	0.0260 (11)	0.0349 (12)	0.0021 (10)	0.0005 (10)	−0.0064 (9)
C2	0.0457 (14)	0.0309 (12)	0.0413 (13)	0.0110 (10)	−0.0078 (11)	−0.0100 (10)
C3	0.0599 (17)	0.0277 (12)	0.0493 (15)	0.0075 (11)	−0.0035 (13)	−0.0115 (11)
C4	0.0610 (17)	0.0320 (12)	0.0430 (14)	−0.0069 (12)	−0.0048 (12)	−0.0102 (11)
C5	0.0442 (15)	0.0409 (14)	0.0579 (16)	0.0037 (12)	−0.0131 (13)	−0.0099 (12)
C6	0.0426 (14)	0.0328 (13)	0.0581 (16)	0.0096 (11)	−0.0066 (12)	−0.0144 (12)
C7	0.0418 (13)	0.0422 (13)	0.0349 (12)	0.0025 (11)	−0.0064 (10)	−0.0163 (11)
C8	0.0531 (15)	0.0386 (13)	0.0346 (12)	0.0026 (11)	−0.0068 (11)	−0.0092 (11)
C9	0.163 (4)	0.0344 (16)	0.061 (2)	−0.001 (2)	−0.026 (2)	−0.0127 (15)
C10	0.072 (2)	0.074 (2)	0.0448 (16)	0.0038 (18)	0.0027 (15)	−0.0050 (15)
C11	0.066 (2)	0.087 (3)	0.060 (2)	0.0057 (19)	−0.0204 (17)	0.0012 (18)
N1	0.0554 (13)	0.0292 (10)	0.0359 (11)	0.0087 (9)	−0.0031 (10)	−0.0133 (9)
O1	0.0609 (12)	0.0500 (11)	0.0493 (11)	0.0138 (9)	−0.0197 (9)	−0.0200 (9)
O2	0.0754 (13)	0.0433 (10)	0.0366 (9)	0.0141 (9)	0.0048 (9)	−0.0110 (8)
O3	0.0613 (12)	0.0543 (12)	0.0489 (11)	0.0205 (10)	0.0008 (9)	−0.0175 (9)
Cl1	0.1118 (8)	0.0485 (5)	0.0812 (6)	−0.0064 (4)	−0.0330 (5)	−0.0266 (4)
S1	0.0536 (4)	0.0328 (3)	0.0340 (3)	0.0090 (3)	−0.0047 (3)	−0.0110 (2)

### Geometric parameters (Å, °)

**Table:**

C1—C6	1.378 (4)	C8—C9	1.520 (4)
C1—C2	1.391 (3)	C8—C10	1.523 (4)
C1—S1	1.763 (3)	C9—H9A	0.9600
C2—C3	1.380 (4)	C9—H9B	0.9600
C2—H2	0.9300	C9—H9C	0.9600
C3—C4	1.374 (4)	C10—H10A	0.9600
C3—H3	0.9300	C10—H10B	0.9600
C4—C5	1.387 (4)	C10—H10C	0.9600
C4—Cl1	1.736 (3)	C11—H11A	0.9600
C5—C6	1.379 (4)	C11—H11B	0.9600
C5—H5	0.9300	C11—H11C	0.9600
C6—H6	0.9300	N1—S1	1.649 (2)
C7—O3	1.208 (3)	N1—H1N	0.82 (3)
C7—N1	1.389 (3)	O1—S1	1.419 (2)
C7—C8	1.525 (3)	O2—S1	1.4354 (19)
C8—C11	1.518 (4)		
			
C6—C1—C2	121.1 (2)	C8—C9—H9A	109.5
C6—C1—S1	119.36 (19)	C8—C9—H9B	109.5
C2—C1—S1	119.5 (2)	H9A—C9—H9B	109.5
C3—C2—C1	119.0 (2)	C8—C9—H9C	109.5
C3—C2—H2	120.5	H9A—C9—H9C	109.5
C1—C2—H2	120.5	H9B—C9—H9C	109.5
C4—C3—C2	119.5 (2)	C8—C10—H10A	109.5
C4—C3—H3	120.2	C8—C10—H10B	109.5
C2—C3—H3	120.2	H10A—C10—H10B	109.5
C3—C4—C5	121.7 (3)	C8—C10—H10C	109.5
C3—C4—Cl1	120.0 (2)	H10A—C10—H10C	109.5
C5—C4—Cl1	118.2 (2)	H10B—C10—H10C	109.5
C6—C5—C4	118.7 (3)	C8—C11—H11A	109.5
C6—C5—H5	120.6	C8—C11—H11B	109.5
C4—C5—H5	120.6	H11A—C11—H11B	109.5
C1—C6—C5	119.8 (2)	C8—C11—H11C	109.5
C1—C6—H6	120.1	H11A—C11—H11C	109.5
C5—C6—H6	120.1	H11B—C11—H11C	109.5
O3—C7—N1	120.3 (2)	C7—N1—S1	123.41 (18)
O3—C7—C8	124.5 (2)	C7—N1—H1N	123 (2)
N1—C7—C8	115.1 (2)	S1—N1—H1N	112 (2)
C11—C8—C9	110.4 (3)	O1—S1—O2	118.95 (12)
C11—C8—C10	109.3 (3)	O1—S1—N1	110.80 (12)
C9—C8—C10	110.1 (3)	O2—S1—N1	103.81 (11)
C11—C8—C7	108.0 (2)	O1—S1—C1	108.91 (12)
C9—C8—C7	110.1 (2)	O2—S1—C1	109.30 (12)
C10—C8—C7	109.0 (2)	N1—S1—C1	103.99 (11)
			
C6—C1—C2—C3	0.3 (4)	O3—C7—C8—C10	7.1 (4)
S1—C1—C2—C3	−178.41 (19)	N1—C7—C8—C10	−175.6 (2)
C1—C2—C3—C4	−0.6 (4)	O3—C7—N1—S1	9.2 (4)
C2—C3—C4—C5	−0.1 (4)	C8—C7—N1—S1	−168.17 (19)
C2—C3—C4—Cl1	179.8 (2)	C7—N1—S1—O1	−57.7 (2)
C3—C4—C5—C6	1.0 (4)	C7—N1—S1—O2	173.5 (2)
Cl1—C4—C5—C6	−178.9 (2)	C7—N1—S1—C1	59.2 (2)
C2—C1—C6—C5	0.6 (4)	C6—C1—S1—O1	170.94 (19)
S1—C1—C6—C5	179.4 (2)	C2—C1—S1—O1	−10.3 (2)
C4—C5—C6—C1	−1.3 (4)	C6—C1—S1—O2	−57.6 (2)
O3—C7—C8—C11	−111.5 (3)	C2—C1—S1—O2	121.2 (2)
N1—C7—C8—C11	65.7 (3)	C6—C1—S1—N1	52.8 (2)
O3—C7—C8—C9	127.9 (3)	C2—C1—S1—N1	−128.5 (2)
N1—C7—C8—C9	−54.8 (3)		

### Hydrogen-bond geometry (Å, °)

**Table:**

*D*—H···*A*	*D*—H	H···*A*	*D*···*A*	*D*—H···*A*
N1—H1N···O2^i^	0.82 (3)	2.19 (3)	2.986 (3)	165 (3)

Symmetry codes: (i) −*x*+1, −*y*, −*z*+1.
